# Supplementary material for: A Comparative Assessment of Approvals and Discontinuations of Systemic Antibiotics and Other Therapeutic Areas
Source: Healthcare (Basel). 2023 Jun 15;11(12):1759. doi: 10.3390/healthcare11121759 (PMC10298669; doi:10.3390/healthcare11121759)
Supplement: Supplementary file 1 [file healthcare-11-01759-s001.zip › healthcare-2318548-supplementary.pdf]

Table S1. FDA Approved New Systemic Antibiotics by Pharmacology Class, 1980-2021

Pharmacology Class	1980-PDUFA	PDUFA-FDAMA	FDAMA-FDASIA	FDASIA- Cures Act	Cures Act-2021	Total
**Aminoglycoside Antibacterials**	**2**				**1**	**3**
Other Aminoglycosides	2				1	3
netilmicin sulfate*	1					1
plazomicin sulfate[#]					1	1
sisomicin sulfate*	1					1
**Beta-Lactam Antibacterials, Penicillins**	**7**	**1**				**8**
Penicillins With Extended Spectrum	5					5
amdinocillin*	1					1
azlocillin sodium*	1					1
bacampicillin hydrochloride*	1					1
mezlocillin sodium monohydrate*	1					1
piperacillin sodium	1					1
Combinations of penicillins, incl. beta-lactamase inhibitors	2	1				3
amoxicillin; clavulanate potassium	1					1
ampicillin sodium; sulbactam sodium	1					1
piperacillin sodium; tazobactam sodium		1				1
**Other Beta-Lactam Antibacterials**	**20**	**3**	**5**	**2**	**3**	**33**
Second-Generation Cephalosporins	8					8
cefmetazole sodium*	1					1
cefonicid sodium*	1					1
ceforanide*	1					1
cefotetan disodium	1					1
cefotiam hydrochloride*	1					1
cefprozil	1					1
cefuroxime sodium	1					1
loracarbef*	1					1
Third-Generation Cephalosporins	10	1	2	1		14
avibactam sodium; ceftazidime[#]				1		1
cefdinir			1			1
cefditoren pivoxil*			1			1
cefixime	1					1
cefmenoxime hydrochloride*	1					1
cefoperazone sodium*	1					1
cefotaxime sodium	1					1
cefpiramide sodium*	1					1
cefpodoxime proxetil	1					1
ceftazidime	1					1
ceftibuten dihydrate*		1				1

ceftizoxime sodium*	1					1
ceftriaxone sodium	1					1
moxalactam disodium*	1					1
Fourth-Generation Cephalosporins		1				1
cefepime hydrochloride		1				1
Carbapenems	1	1	2		2	6
cilastatin sodium; imipenem	1					1
cilastatin sodium; imipenem; relebactam[#]					1	1
doripenem*			1			1
ertapenem sodium			1			1
meropenem		1				1
meropenem; vaborbactam[#]					1	1
Monobactams	1					1
aztreonam	1					1
Other Cephalosporins and Penems			1	1	1	3
cefiderocol sulfate tosylate[#]					1	1
ceftaroline fosamil			1			1
ceftolozane sulfate; tazobactam sodium[#]				1		1
**Macrolides, Lincosamides and Streptogramins**	**2**	**1**	**2**			**5**
Macrolides	2	1	1			4
azithromycin	1					1
clarithromycin	1					1
dirithromycin*		1				1
telithromycin*			1			1
Streptogramins			1			1
dalfopristin; quinupristin			1			1
**Quinolone Antibacterials**	**7**	**2**	**5**		**1**	**15**
Fluoroquinolones	6	2	5		1	14
alatrofloxacin mesylate[&]			1			1
ciprofloxacin hydrochloride	1					1
delafloxacin meglumine[#]					1	1
enoxacin*	1					1
gatifloxacin[&]			1			1
gemifloxacin mesylate*			1			1
grepafloxacin hydrochloride&		1				1
lomefloxacin hydrochloride*	1					1
moxifloxacin hydrochloride			1			1
norfloxacin*	1					1
ofloxacin	1					1
Sparfloxacin[&]		1				1

temafloxacin hydrochloride[&]	1					1
trovafloxacin mesylate[&]			1			1
Other Quinolones	1					1
cinoxacin*	1					1
**Tetracyclines**			**1**		**3**	**4**
eravacycline dihydrochloride[#]					1	1
omadacycline tosylate[#]					1	1
sarecycline hydrochloride					1	1
tigecycline			1			1
**Other Antibacterials**		**1**	**3**	**3**	**2**	**9**
Glycopeptide Antibacterials			1	2		3
dalbavancin hydrochloride[#]				1		1
oritavancin diphosphate[#]				1		1
telavancin hydrochloride			1			1
Imidazole Derivatives					1	1
secnidazole[#]					1	1
Other Antibacterials		1	2	1	1	5
daptomycin			1			1
fosfomycin tromethamine		1				1
lefamulin acetate[#]					1	1
linezolid			1			1
tedizolid phosphate[#]				1		1
**Total**	**38**	**8**	**16**	**5**	**10**	**77**

*Market discontinuation. [&]Withdrawal of approval for safety reasons. [#]Qualified Infectious Disease Product -QIDP.

Table 2. Indications of FDA Approved New Antibiotics, 1980-2021

	1980-PDUFA	PDUFA-FDAMA	FDAMA-FDASIA	FDASIA-Cures Act	Cures Act-2021	Total
**Skin and skin structure infections**	**25**	**4**	**16**	**3**	**2**	**50**
Complicated		1	9			10
Uncomplicated	5	2	5			12
Uncomplicated and complicated		1				1
Not specified	20		2	3	2	27
**Urinary tract infections**	**36**	**2**	**4**	**2**	**4**	**48**
Complicated	6		3	1	4	14
Complicated and Uncomplicated	4	1		1		6
Uncomplicated	3	1	1			5
Not specified	23					23
**Pyelonephritis**	**1**		**1**			**2**
Uncomplicated	1					1
Not specified			1			1
**Intra-abdominal infections**	**14**	**1**	**6**	**2**	**2**	**25**
Complicated	1	1	6	2	2	12
Not specified	13					13
**Appendicitis and Peritonitis**		**2**				**2**
Complicated		2				2
**Community Acquired Pneumonia**	**5**	**5**	**12**		**3**	**25**
Not specified	5	5	12		3	25
**Hospital-acquired Bacterial Pneumonia/Ventilator-associated Bacterial Pneumonia**			**1**	**2**	**2**	**5**
Not specified			1	2	2	5
**Nosocomial Pneumonia**		**1**	**3**			**4**
Not specified		1	3			4
**Lower Respiratory Tract Infections**	**17**	**1**				**18**
Not specified	17	1				18
**Bronchitis, Chronic**	**7**	**4**	**6**			**17**
Uncomplicated	1					1
Not specified	6	4	6			16
**Bronchitis, Acute**	**2**					**2**
Not specified	2					2
**Respiratory Tract Infections**	**2**					**2**

Not specified	2					2
**Upper respiratory tract infections**	**1**	**1**				**2**
Not specified	1	1				2
**Gonorrhea**	**15**	**1**	**1**			**17**
Uncomplicated	15	1	1			17
**Gynecologic Infections**	**11**		**2**			**13**
Not specified	11		2			13
**Pelvic inflammatory disease**	**4**		**1**			**5**
Not specified	4		1			5
**Pelvic Infections**	**1**		**1**			**2**
Not specified	1		1			2
**Postpartum Endometritis/Pelvic Inflammatory Disease**		**1**				**1**
Not specified		1				1
**Urethritis and Cervicitis**			**1**			**1**
Not specified			1			1
**Chlamydia Trachomatis**	**1**					**1**
Not specified	1					1
**Genital Infections**	**1**					**1**
Not specified	1					1
**Bone/Joint Infections**	**13**					**13**
Not specified	13					13
**Septicemia**	**12**					**12**
Not specified	12					12
**Prophylaxis, Surgical**	**6**		**2**			**8**
Not specified	6		2			8
**Pharyngitis/Tonsillitis**	**5**	**1**	**2**			**8**
Not specified	5	1	2			8
**Meningitis**	**5**	**2**				**7**
Not specified	5	2				7
**Otitis Media**	**5**	**1**	**1**			**7**
Not specified	5	1	1			7
**Sinusitis**	**3**		**3**			**6**
Not specified	3		3			6
**Maxillary sinusitis**	**3**		**1**			**4**

Not specified	3		1			4
**Prostatitis**	**3**					**3**
Not specified	3					3
**Nongonococcal Urethritis/Cervicitis**	**2**	**1**				**3**
Not specified	2	1				3
**Central Nervous System Infections**	**2**					**2**
Not specified	2					2
**Bacteremia**			**2**			**2**
Not specified			2			2
**Bacteremia/Septicemia**	**2**					**2**
Not specified	2					2
**Vancomycin-Resistant Enterococcus faecium infections**			**2**			**2**
Not specified			2			2
**Endocarditis**	**2**					**2**
Not specified	2					2
**Pseudomonas Aeruginosa Infections**	**1**					**1**
Not specified	1					1
**Enterococcal Infections**	**1**					**1**
Not specified	1					1
**Pseudomonas Aeruginosa/Escherichia Coli/Haemophilus Influenzae Infections**	**1**					**1**
Not specified	1					1
**Infections Caused by Malignant Diseases**	**1**					**1**
Not specified	1					1
**Total**	**210**	**28**	**68**	**9**	**13**	**328**
